# Tele-supervised home-based transcranial alternating current stimulation (tACS) for Alzheimer’s disease: a pilot study

**DOI:** 10.3389/fnhum.2023.1168673

**Published:** 2023-06-02

**Authors:** Davide Cappon, Rachel Fox, Tim den Boer, Wanting Yu, Nicole LaGanke, Gabriele Cattaneo, Ruben Perellón-Alfonso, David Bartrés-Faz, Brad Manor, Alvaro Pascual-Leone

**Affiliations:** ^1^Hinda and Arthur Marcus Institute for Aging Research at Hebrew SeniorLife, Boston, MA, United States; ^2^Deanna and Sidney Wolk Center for Memory Health at Hebrew SeniorLife, Boston, MA, United States; ^3^Department of Neurology, Harvard Medical School, Boston, MA, United States; ^4^Institut Guttmann, Institut Universitari de Neurorehabilitació Adscrit a la UAB, Barcelona, Spain; ^5^Departament de Medicina, Universitat Autònoma de Barcelona, Bellaterra, Spain; ^6^Fundació Institut d’Investigació en Ciències de la Salut Germans Trias i Pujol, Badalona, Spain; ^7^Department of Medicine, Faculty of Medicine and Health Sciences, Institute of Neurosciences, University of Barcelona, Barcelona, Spain; ^8^Institute of Biomedical Research August Pi i Sunyer (IDIBAPS), Barcelona, Spain; ^9^Department of Medicine, Harvard Medical School, Boston, MA, United States

**Keywords:** Alzheimer’s disease, neuromodulation, transcranial alternating current stimulation (tACS), memory, cognition, electroencephalography (EEG)

## Abstract

**Background:**

Over 55 million people worldwide are currently diagnosed with Alzheimer’s disease (AD) and live with debilitating episodic memory deficits. Current pharmacological treatments have limited efficacy. Recently, transcranial alternating current stimulation (tACS) has shown memory improvement in AD by normalizing high-frequency neuronal activity. Here we investigate the feasibility, safety, and preliminary effects on episodic memory of an innovative protocol where tACS is administered within the homes of older adults with AD with the help of a study companion (HB-tACS).

**Methods:**

Eight participants diagnosed with AD underwent multiple consecutive sessions of high-definition HB-tACS (40 Hz, 20-min) targeting the left angular gyrus (AG), a key node of the memory network. The Acute Phase comprised 14-weeks of HB-tACS with at least five sessions per week. Three participants underwent resting state electroencephalography (EEG) before and after the 14-week Acute Phase. Subsequently, participants completed a 2–3-month Hiatus Phase not receiving HB-tACS. Finally, in the Taper phase, participants received 2–3 sessions per week over 3-months. Primary outcomes were safety, as determined by the reporting of side effects and adverse events, and feasibility, as determined by adherence and compliance with the study protocol. Primary clinical outcomes were memory and global cognition, measured with the Memory Index Score (MIS) and Montreal Cognitive Assessment (MoCA), respectively. Secondary outcome was EEG theta/gamma ratio. Results reported as mean ± SD.

**Results:**

All participants completed the study, with an average of 97 HB-tACS sessions completed by each participant; reporting mild side effects during 25% of sessions, moderate during 5%, and severe during 1%. Acute Phase adherence was 98 ± 6.8% and Taper phase was 125 ± 22.3% (rates over 100% indicates participants completed more than the minimum of 2/week). After the Acute Phase, all participants showed memory improvement, MIS of 7.25 ± 3.77, sustained during Hiatus 7.00 ± 4.90 and Taper 4.63 ± 2.39 Phases compared to baseline. For the three participants that underwent EEG, a decreased theta/gamma ratio in AG was observed. Conversely, participants did not show improvement in the MoCA, 1.13 ± 3.80 after the Acute Phase, and there was a modest decrease during the Hiatus −0.64 ± 3.28 and Taper −2.56 ± 5.03 Phases.

**Conclusion:**

This pilot study shows that the home-based, remotely-supervised, study companion administered, multi-channel tACS protocol for older adults with AD was feasible and safe. Further, targeting the left AG, memory in this sample was improved. These are preliminary results that warrant larger more definite trials to further elucidate tolerability and efficacy of the HB-tACS intervention. NCT04783350.

**Clinical trial registration:**

https://clinicaltrials.gov/ct2/show/NCT04783350?term=NCT04783350&draw=2&rank=1, identifier NCT04783350.

## Introduction

Over 55 million people worldwide are currently diagnosed with Alzheimer’s disease (AD) and this number is predicted to nearly double by the year 2050, due to the aging population (Alzheimer’s Association, 2022; World Health Organization, 2022). Early on in the course of their condition, AD patients typically have episodic memory loss, which is devastating to them as well as to their family and caregivers and is a useful indicator of how the disease will progress (El Haj et al., 2017; Abraham et al., 2020).

The targets of current pharmacological therapy for memory enhancement are beta-amyloid plaque (β-amyloid) deposition (Avgerinos et al., 2021; van Dyck et al., 2022), one of the pathophysiological hallmarks of AD, and the related dysregulation of the cholinergic system (Hampel et al., 2018). Alongside cholinergic dysregulation and β-amyloid accumulation, recent studies have shown that high frequency brain network activities that support successful memory encoding and recall of new information are altered decades before AD clinical onset, and the disrupted networks predict future pathology and brain atrophy (Koenig et al., 2005; Sperling et al., 2009; Babiloni et al., 2016; Palop and Mucke, 2016; Palop, 2020). Specifically, recent research has focused primarily on the role of gamma desynchronization (30–80 Hz) in AD manifestation, raising the possibility of exploiting it as a novel therapeutic target.

In animal models of AD, entrainment-induced restoration of gamma oscillations reduces the pathogenic load of β-amyloid and significantly improves behavior (Iaccarino et al., 2016; Adaikkan et al., 2019; Etter et al., 2019; Martorell et al., 2019). Further, neuroimaging evidence indicate that the AG is a key node in the memory network, and gray matter volume reduction in the AG has been linked to AD memory symptoms (Oh et al., 2013; Humphreys et al., 2021; van de Mortel et al., 2021). For the translation of these findings to humans, transcranial alternating current stimulation (tACS) has drawn interest for its ability to modulate cortical excitability and improve cognitive functions by safely modulating brain activity at a precise frequency in targeted brain structures (Zaehle et al., 2010; Antal and Paulus, 2013; Herrmann et al., 2013; Cappon et al., 2015, 2016). Recent initial studies in AD have demonstrated that the application of tACS at gamma frequency targeting key nodes of the memory network improved episodic memory and restored cholinergic neurotransmission (Benussi et al., 2021; Kim et al., 2021; Benussi et al., 2022; Zhou et al., 2022).

These recent findings are very encouraging for the application of tACS as a safe and effective intervention in patients with AD. However, studies to date have been limited by the fact that only a single tACS session was administered in a clinic-lab setting, which raises questions about how long memory effects will last and whether it will be possible to scale up tACS interventions for patients who cannot afford to travel to a specialized clinic center. Thus, there is an urgent need for a safe, effective, and more accessible home-based intervention for memory in AD.

We have previously developed an innovative methodology and demonstrated the safety and feasibility of providing a home-based, remotely supervised, study companion (e.g., caregiver, family member, friend) administered, multi-channel neuromodulation protocol for older adults (Cappon et al., 2022). This approach offers the opportunity to integrate therapeutic benefit into the daily life of participants and increasing access to tACS treatment. Caregivers are also empowered as they play a vital role as study companions directly assisting in treatment for their loved one. In the present pilot study, we report the results of a case series investigation including eight AD-diagnosed participants who received multiple sessions of home-based, remotely supervised, study companion administered, multi-channel tACS targeted at the left angular gyrus (L-AG). Assessing the intervention’s safety, feasibility, and early clinical impact on episodic memory were the study’s primary objectives.

## Materials and methods

### Participants

Ten participants were enrolled in the study, with two participants (P004 and P009) withdrawing due to personal reasons unrelated to their participation in the trial. Eight participants diagnosed with AD received home-based transcranial alternating current stimulation (HB-tACS) from March 3rd, 2021 to June 3rd, 2022. Demographic and clinical characteristics of participants are presented in Table 1. All participants had amnestic impairment, verified by the study neurologist. Inclusion criteria for participants were: AD diagnosis (characterized by AD biomarker positivity and by in-person assessment by qualified health care personnel), over the age of 50, able to give consent, ability to read, write, and communicate in English, and able to identify an eligible study companion to participate with them in the study to administer the HB-tACS. Exclusion criteria included major psychiatric co-morbidities, dermatological conditions on the scalp, an inability to provide informed consent, or any contraindications to tACS (such as recent seizures, implanted medical devices, use of neuroactive drugs, etc.). Inclusion criteria for study companions were: at least 21 years old, self-reported computer proficiency, able to read, write, and communicate in English, willingness to learn how to administer HB-tACS, availability during weekdays of the study period to administer HB-tACS to the study participant, and a MoCA score of 27–30 to demonstrate their cognitive capacity. Study companions demographic characteristics and the number of HB-tACS sessions completed during the study are displayed in Table 2.

**TABLE 1:** Participant demographics and clinical characteristics.

Participant	Age	Sex	Education (years)	Diagnosis	MoCA score (Baseline)	Memory Index Score (Baseline)
P001	79	M	19	Late onset AD	21	0
P002	79	M	19	Late onset AD	23	0
P003	88	F	16	AD	28	0
P006	79	F	16	AD	17	1
P007	71	M	18	AD	18	0
P008	66	M	16	Moderate AD	10*	1
P010	53	M	16	Early onset moderate AD	10*	0
P011	76	M	16	AD	22	4

*Please note P008 and P010 were more impaired in their baseline MoCA score, but as this is a pilot study, we sought to be more inclusive of our participants and their level of decline.

**TABLE 2:** Study companions demographic, level of education, and the number of sessions completed.

Study companion for	Age	Sex	Education (years)	Number of HB-tACS sessions completed	
				**Acute Phase**	**Taper Phase**
P001	78	F	16	70	48
P002	73	F	16	69	35
P003	88	M	18	64	20
P006	80	M	16	69	22
P007	64	F	16	79	24
P008	59	F	16	64	24
P010	53	F	16	68	26
P011	74	F	16	66	32

### Study design

**FIGURE 1:**
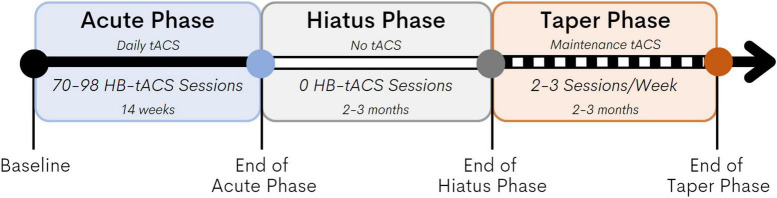
Study design. The Acute Phase lasted 14 weeks, the study companions conducted daily 20-min HB-tACS sessions with a minimum of five sessions per week (maximum of one session per day, seven sessions per week). The Hiatus lasted 3 months no HB-tACS sessions were administered. In the Taper Phase, we asked participants to complete 2–3 HB-tACS sessions per week over 3 months.

The study intervention consisted of multiple consecutive sessions of HB-tACS (40 Hz, 20-min) targeting the left angular gyrus (L-AG), a key node of the memory network. The protocol consisted of three phases: Acute Phase, Hiatus Phase, and the Taper Phase (see Figure 1). During the Acute Phase, the study companions conducted daily 20-min HB-tACS sessions for 14 weeks, with a minimum of five sessions each week (maximum of one session per day, seven sessions per week). A subset of three participants completed an optional resting state high-density electroencephalography (HD-EEG) visit at baseline and at the end of the acute phase. HD-EEG was included to gain insight into the underlying neurophysiological alterations induced by tACS, we opted to make it optional to minimize the burden of additional assessments since it wasn’t a primary outcome of the present study. After the Acute Phase, participants and their study companions completed 3 months without any stimulation during the Hiatus Phase. The goal of the Hiatus Phase was to capture the extent to which any effects of HB-tACS on memory may have been sustained after 3 months without stimulation. Subsequently, in the Taper phase, we asked participants to complete 2–3 HB-tACS sessions per week over 3 months. The objective of this phase was to facilitate maintenance of any benefit derived from HB-tACS in the Acute Phase. Assessments were conducted at baseline and at the end of each of the three phases. Further, we monitored adherence to the HB-tACS protocol through all phases and a member of the research staff called the study companions once a week during the Acute and the Taper Phase to check on the participants’ condition and to record any subjective effects of tACS on their memory.

### Study procedures

The study was approved and monitored by an independent Institutional Review Board (Advarra, Inc.) and registered as clinical trial at clinicaltrials.gov (NCT04783350). All subject interaction was conducted either in-person at the Hebrew Rehabilitation Center in Boston, MA, or remotely via videoconferencing media. Prior to enrolling in the study, participants and study companions attended a 45-min phone screening to ensure they fulfilled eligibility criteria. If eligible, participants then completed an in-person screening visit comprised of a medication history, neurological exam, Clinical Dementia Rating (CDR), and a Montreal Cognitive Assessment (MoCA) (from which the Memory Index Score (MIS) is calculated) to assess global cognitive status. Participants were given the choice to undergo resting state HD-EEG recording if they were eligible for the study.

### Optimized tACS current flow modeling to target the left angular gyrus

**FIGURE 2:**
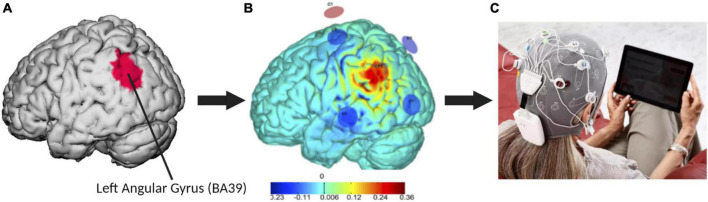
The multichannel tACS intervention for AD. **(A)** The red area on the default brain model represents the left angular gyrus, or Brodmann’s area 39 (BA39). This target area was used to develop the current flow modeling and electrode montage. **(B)** The optimized six-electrode montage developed in this study to target the left AG (anode shown in red, cathodes in blue) and normal component of the electric field to the cortex induced by the montage (V/m). **(C)** Set-up of the Neuroelectrics Starstim^®^ Home Kit headcap and tablet.

The specific tACS system used in this study is the Starstim^®^-Home Kit (Neuroelectrics Corp.). The Starstim^®^ device includes a headcap that resembles a swimming cap with holes located where small electrodes can be attached and secured in place in the correct position on the scalp. These electrode holes are color- and number-coded so that electrode leads with corresponding colors coming from the tACS device are appropriately attached to the corresponding electrodes, eliminating the potential for accidental mismatching of the electrodes and the leads. Each session involved a multichannel tACS montage with maximal anodal current targeting the left AG administered via 6 NG Pistim electrodes (circular electrodes with a contact of area of 3.14 cm^2^) using the Starstim^®^-Home system. Created using the Stimweaver^®^ algorithm (Ruffini et al., 2014), the montage was specifically designed to optimize stimulation over the left AG and at the same time minimize off target stimulation effects based on a standard brain model (see Figure 2). During each sessions the currents delivered by any single electrode did not exceed 2.0 mA, well below recommended safety limits (Antal et al., 2017). The average E-field normal component on the target was En = 0.24 V/m, and En = 0.10 V/m on the surrounding regions. For all participants, current intensity was ramped up over 60 s, then sustained at the stimulation intensity for 20 min, then ramped down over 60 s. This standard approach is both well-tolerated and safe in older adults (Cappon et al., 2022).

### Home-based study companion-led tACS administration

After being enrolled, participants and their study companions came to the laboratory on three consecutive days to receive hands-on instruction in the administration of tACS. On the first day, study companions were given an overview of the Starstim^®^-Home Kit (Neuroelectrics Corp.) tACS equipment by a trained research staff member. On the same day, research staff administered the first tACS session to the study participant, instructing the study companion who observed the administration. On the second day, the study companion was encouraged to carry out as much of the tACS session as possible without guidance from the research staff. However, the study companion was allowed to ask for assistance as necessary, and research staff corrected them if they made any mistakes. On the third day, study companions independently administered the stimulation to the participant, while research staff assessed the performance of the study companion using an evaluation check list adapted from Charvet et al. (2015). Participants then brought the HB-tACS device home to complete stimulation sessions independently with remote supervision from research staff. For further explanation of this HB-tES training and supervision program, please see Cappon et al. (2022) and den Boer et al. (2022).

### Study outcomes

#### Safety

##### Side effects questionnaire and adverse events

Before and after each home-based tACS session, participants were asked to report any side effects on questionnaires that were implemented using the Starstim^®^-Home system smart tablet. Based on previous published safety guidelines, participants were asked to report on any physical sensations, scalp abnormalities, cognitive changes, or other symptoms that were present (Antal et al., 2017). A list of the side effects that were asked about can be found in Table 3. Participants were asked to report on the intensity of the side effects, by stating whether their experience for each of the side effects was: absent, mild, moderate, or severe. These responses were received via email by research staff through a study email inbox in real-time. If participants indicated a moderate or severe experience of a side effect prior to the tACS, access to the stimulation was blocked until study staff could ensure that the participant was fit for stimulation.

**TABLE 3:** Total incidence of side effects and their severity inquired about in the side effect questionnaires pre and post tACS.

Side effect	Mild		Moderate		Severe		Percentage of sessions
	**Pre**	**Post**	**Pre**	**Post**	**Pre**	**Post**	
Headache	0	0	0	0	0	0	0%
Neck pain	0	2	0	0	0	0	0.2%
Scalp pain	0	2	1	2	0	1	0.81%
Scalp burns	3	1	0	2	0	1	0.9%
Sensations under the electrode	3	162*	1	27	0	6	25.6%
Skin redness	1	3	0	0	0	0	0.5%
Sleepiness	1	18	0	11	0	1	4%
Trouble concentrating	0	9	0	0	0	0	1.2%
Acute mood change	1	1	0	0	0	0	0.2%
Other	0	0	0	0	0	0	0%
Total %	1.2%	25.4%	0.2%	5.4%	0%	1.2%	33.4%

*P007 and P010 reported the highest frequency of “sensations under the electrode”, accounting for over 79% of reports (see Table 4).

#### Feasibility

##### Adherence and compliance

The adherence to the treatment schedule, measured as the ratio of completed HB-tACS sessions to the required sessions as prescribed in the study schedule (a minimum of five sessions per week in the Acute Phase, and a minimum of two sessions per week in the Taper Phase), was used to evaluate the feasibility of the home-based tDCS protocol. This allowed us to capture how HB-tACS session administration can fit into the daily life of the participant and study companion and help determine if this method is feasible for future use in an older adult population.

##### Clinical outcomes

Clinical outcomes focused on memory and global cognitive functioning, as measured by the Memory Index Score (MIS) and the full Montreal Cognitive Assessment (MoCA), respectively. Research staff who have completed MoCA training and obtained certification administered these examinations to ensure consistency in the administration. They were assessed at baseline, after the Acute Phase, after the Hiatus Phase, and after the Taper Phase. At the end of each of the three phases, data were acquired and stored using a REDCap database, a secure platform for storing data and generating reports.

##### Montreal cognitive assessment (MoCA)

The MoCA is a clinician-rated screening test consisting of 12 items across different cognitive domains, including visuospatial/executive functioning, animal naming, memory, attention, language, abstraction, delayed recall, and orientation. It can be administered within 10 min, resulting in a maximum total score of 30 points. Lower scores indicate greater cognitive decline, with MoCA scores below 26 indicating the presence of mild cognitive decline (Nasreddine et al., 2005). The 5-min telehealth MoCA was used over the telephone or videoconferencing media as a way to assess cognition without requiring the participant to travel to the research laboratory (Wong et al., 2015). The MoCA 5-min is comprised of five sub-tests that were taken from the MoCA and look at different cognitive domains: orientation, executive functions/language, verbal learning and memory, and attention. This shortened 5-min MoCA is highly correlated in score with the full 12-item MoCA (Wong et al., 2015). The version of the MoCA (including MIS words) was randomized for each assessment to minimize practice effects, though we acknowledge that there is still a potential impact of practice effects (Lei et al., 2022).

##### Memory Index Score (MIS)

The MIS is a measure of delayed recall memory that is calculated from the MoCA (either the full or 5-min). Five unrelated words are spoken to the participant twice, and each time they are repeated by the participant to facilitate the encoding process. After a 5-min delay, participants are asked to recall the five words. If freely recalled without a cue, 3 points per word is earned toward the MIS. If recalled with a categorical cue, 2 points per word is earned. If recalled when presented with multiple choice options, 1 point per word is earned, for a maximum possible score of 15 points (Julayanont et al., 2014). Thus, the MIS score computation includes points for both the free recall and cued recall conditions, unlike the MoCA, which only accounts for points for the delayed free recall condition. Due to how the MIS is calculated, a large effect can be seen in a participant’s MIS score, but only minimal changes seen in the MoCA overall score.

#### EEG recording and analysis

For those participants who opted in to this part of the study, 5 min of eyes closed resting state high density EEG were recorded continuously using a 256 channels HydroCel Geodesic Sensor Net (Electrical Geodesic Inc., Eugene, OR, USA) at 1 KHz sampling rate and bandpass filtered 0.3 to 500 Hz. Electrode impedance was kept under 50 Kohm. EEG data was pre-processed using EEGLAB (Delorme and Makeig, 2004) and custom-made Matlab (The MathWorks, Inc., Natick, MA, USA) scripts. First, face and neck electrodes were removed from the data. Then we retained only 62 of the remaining scalp electrodes that could be directly mapped to the 10–10 international electrode position system (Luu and Ferree, 2005). Line noise (i.e., 60 Hz and harmonics) was then attenuated using the Zapline algorithm (de Cheveigné, 2020). Excessively noisy or disconnected electrode channels were spline interpolated and data was re-referenced to the average of all channels. Next, the continuous data was segmented into non-overlapping two-second-long epochs. All epochs were visually inspected and those that contained any artifacts were removed. The cleaned preprocessed data was then used for source reconstruction in Brainstorm (Tadel et al., 2019). For each subject a forward model was estimated via the openMEEG algorithm (Kybic et al., 2005) using the default settings (i.e., 3 layers with 1,922 vertices each; skull and scalp conductivities of 1 and brain conductivity of 0.0125; adaptative integration), and based on the MNI ICBM152 average brain template (Mazziotta et al., 1995). The inverse solution was estimated using the minimum norm imaging method (Salmelin and Baillet, 2009). Sources were then computed as current density maps for constrained orientations only (i.e., normal to cortex). We aimed to determine how much the HB-tACS intervention impacted the oscillatory power of gamma oscillations in the left angular gyrus. In source space, we then defined a single region of interest (ROI) containing the left angular gyrus, based on the Desikan-Kiliani cortical parcellation (Desikan et al., 2006). Power spectral density estimates were then extracted from all voxels of this ROI using the Welch method (Welch, 1967) and averaged. Finally, we computed relative power frequency bands for theta (4–7.9 Hz), alpha (8–13.9 Hz), beta (14–29.9 Hz), and gamma (30–50 Hz), and the ratio of theta to gamma.

## Results

### Safety

#### Side effects questionnaire and adverse events

All side effects reported from baseline to the end of the Taper Phase were transient. The most frequently reported side effects were mild and reported during less than 1% of sessions, as shown in Table 3. The two exceptions to this, however, were the side effects “Sensations under the electrode” and “Sleepiness.” Participants reported sensations under the electrode (such as a tingling or an itching feeling) during 25.6% of sessions. Feeling sleepy as a consequence of the stimulation was reported during 4% of sessions, and found to be transient (Table 4). Overall, side effects were reported in 33.4% of sessions, with participants reporting mild side effects during 26.6% of sessions, moderate side effects during 5.6% of sessions, and severe side effects during 1.2% of sessions as seen in Table 3. Upon the participants reporting a moderate or severe side effect, research staff was notified and would immediately contact participants and gather more information about the side effect and how they were currently feeling. After this contact, research staff would report the situation to the medically responsible study physician, who would assess the event and advise participants on how to proceed. The monitoring of adverse events showed that no adverse events occurred during the course of the study.

**TABLE 4:** Frequency with which participants reported sensations under the electrodes.

Participant	# Reported sensations under the electrodes
P001	4
P002	1
P003	8
P006	46
P007	63
P008	1
P010	65
P011	11

#### Feasibility

##### Adherence

All eight participants and their study companions had very high adherence to our protocol, collectively completing 780 HB-tACS sessions between baseline and the end of the Taper Phase. The adherence of individual participants is shown in Table 5. The average percent adherence to the tACS protocol for the Acute Phase was 98% ± 6.8%, showing that participants and their study companions were able to strongly comply with daily tACS. The average adherence in the Taper Phase is encouraging, as when participants were empowered to choose the number of tACS sessions they completed, nearly all completed more than the minimum of 2 sessions per week, with an average adherence of 125% ± 22.3%, or completing an average of 2.5 sessions each week. Adherence rates over 100% indicates participants completed more than the minimum required sessions per week.

**TABLE 5:** Participant adherence to tACS protocol in the Acute Phase and Taper Phase.

Participant	Acute Phase adherence % (minimum 5 sessions/week)	Taper Phase adherence % (minimum 2 sessions/week)
P001	100%	160%*
P002	99%	138%*
P003	91%	91%
P006	99%	100%
P007	113%*	123%*
P008	91%	133%*
P010	97%	139%*
P011	94%	118%*
Average	98% (6.8)	125% (22.3)

*Adherence over 100% indicates participants completed more than the minimum required sessions per week.

#### Cognitive outcomes

##### Memory Index Score (MIS)

**FIGURE 3:**
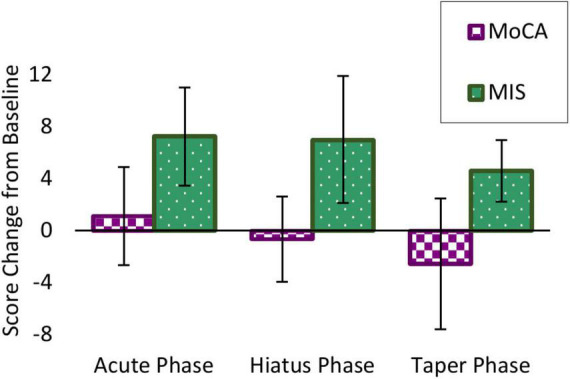
Participant average score change from baseline on Memory Index Score (MIS) (green) and MoCA (purple). While global cognition measured by the MoCA stayed stable with a modest decrease over time, episodic memory measured by the MIS showed great improvement from baseline after the Acute Phase, which was sustained even after the Taper Phase.

**FIGURE 4:**
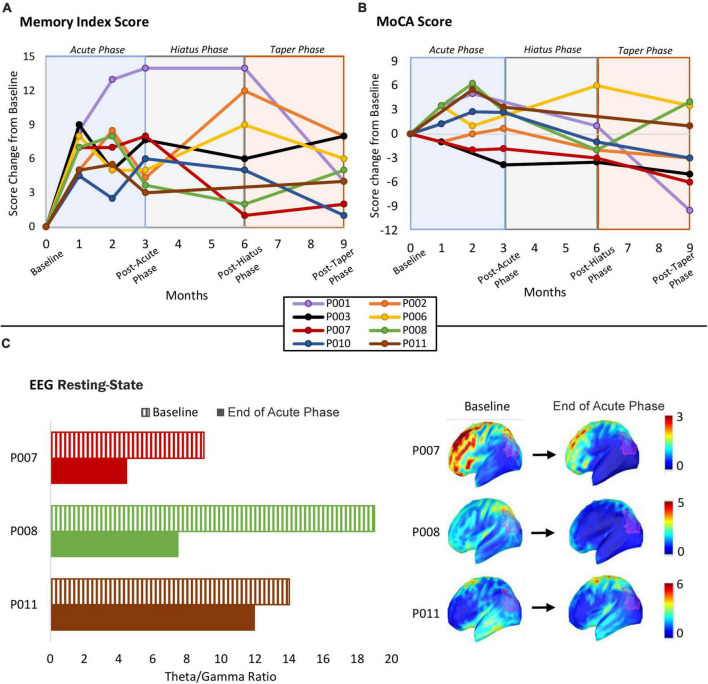
**(A)** Individual participant MIS scores over the course of the study duration. **(B)** Individual participant MoCA scores over the course of the study duration. Shortened 5-min MoCA were administered via videoconferencing media to assess potential short-term effects of the stimulation on global cognitive status and any incremental change at 1 and 2 months of the Acute Phase. **(C)** EEG results: theta/gamma ratio at baseline, and after the Acute Phase of tACS intervention course at the end of Acute Phase. Left panel shows a bar graph of each participant’s theta/gamma ratio at Baseline and at the end of the Acute Phase within the defined left angular gyrus ROI (see in Methods; EEG recording and analysis section). Right panel shows the distribution of theta/gamma ratio in source space and projected onto the MNI template; the red transparent contour highlights the ROI from which the bar graph data was extracted.

All participants showed memory improvement at the end of the Acute Phase, with an average improvement of 7.25 ± 3.77 points. This was sustained during the Hiatus 7.00 ± 4.90 and Taper 4.63 ± 2.39 phases compared to baseline. Even at the end of the study period, all participants had maintained some of the original benefit from the Acute Phase. These average changes in scores are showed in Figure 3, with individual scores in Figure 4A. The individual MIS scores at each assessment point are also displayed in Table 6.

**TABLE 6:** Memory Index Scores (MIS) for all participants and comparisons to baseline.

Participant	Baseline	Acute Phase month 1		Acute Phase month 2		Post-acute phase		Post-hiatus phase		Post-taper phase	
	**Score**	**Score**	**Score change from baseline**	**Score**	**Score change from baseline**	**Score**	**Score change from baseline**	**Score**	**Score change from baseline**	**Score**	**Score change from baseline**
P001	0	8.5	+ 8.5	13	+13	14	+ 14	14	+ 14	4	+4
P002	0	5	+ 5	8.5	+ 8.5	11	+ 11	12	+ 12	8	+8
P003	0	9	+ 9	5	+5	6	+ 6	6	+6	7	+ 7
P006	1	9	+ 8	6	+5	6	+ 5	10	+ 9	7	+6
P007	0	7	+ 7	7	+7	8	+ 8	1	+1	2	+ 2
P008	1	8	+ 7	9	+8	6	+ 5	3	+2	6	+ 5
P010	0	4.5	+ 4.5	2.5	+ 2.5	7	+7	5	+ 5	1	+1
P011	4	9	+ 5	9.5	+ 5.5	6	+2	N/A	N/A	8	+ 4

##### Montreal Cognitive Assessment (MoCA)

Participants did not show any dramatic change in the MoCA at the end of the Acute Phase, with an average of 1.13 ± 3.80 points. There was a modest decrease during the Hiatus −0.64 ± 3.28 and Taper −2.56 ± 5.03 Phases from baseline. These average changes in scores are showed in Figure 3, with individual scores in Figure 4B. The individual MoCA scores at each assessment point are also displayed in Table 7.

**TABLE 7:** MoCA scores for all participants during the course of the study and comparisons to baseline.

Participant	Baseline	Acute Phase month 1		Acute Phase month 2		Post-acute phase		Post-hiatus phase		Post-taper phase	
	**Score**	**Score**	**Score change from baseline**	**Score**	**Score change from baseline**	**Score**	**Score change from baseline**	**Score**	**Score change from baseline**	**Score**	**Score change from baseline**
P001	21	24.5	+ 3.5	26	+5	27	+ 6	22	+ 1	11.5	−9.5
P002	23	22	−1	23	+ 0	25	+ 2	21	−2	20	−3
P003	28	27	−1	23	−5	23	−5	24.5	−3.5	20.5	−7.5
P006	17	20.5	+ 3.5	18	+1	16	−1	23	+ 6	20.5	+ 3.5
P007	18	15	−3	16	−2	15	−3	15	−3	12	−6
P008	10	13.5	+ 3.5	16.5	+ 6.5	14	+ 4	8	−2	14	+ 4
P010	10	11.5	+ 1.5	12.5	+ 2.5	14	+ 4	9	−1	7	−3
P011	22	24	+ 2	27.5	+ 5.5	24	+ 2	N/A	N/A	23	+ 1

#### Neurophysiological outcome

##### EEG resting-state

As the EEG was optional in this study, only a subset of three participants chose to take part: P007, P008, and P011. Theta/gamma ratio, which was assessed in source space within the ROI including the left angular gyrus (L-AG), decreased in all 3 participants after the Acute Phase (Figure 4C).

## Discussion

In the present study, we adopted our recently developed method of providing a home-based, remotely supervised, study companion (e.g., caregiver, family member, friend) administered, multi-channel neuromodulation protocol for older adults (Cappon et al., 2022).

We monitored side effects throughout the experiment using a customized side-effect questionnaire administered on a tablet. No serious adverse events occurred across the total of 780 HB-tACS sessions. The side effects recorded during the study were mostly mild and transient. Specifically, of the 31% of sessions in which side effects were reported, participants reported 80% mild, 17% moderate and 3% severe side effects on an intensity rating with a range of mild, moderate, and severe options. This is in line with previous research that showed that tACS was well-tolerated and that no serious adverse events were reported when tACS was administered in accordance with the safety guidelines (Antal et al., 2017).

We found that the study companions effectively conducted the tACS sessions at home after receiving instruction and training. This was demonstrated by the fact that, on average, 68.6 tACS sessions in the Acute Phase and 28.9 sessions in the Taper Phase were delivered successfully by study companions. This is promising for future research as it shows that the training in tACS administration can empower study companions to conduct the stimulation sessions themselves in the home of the study participants and that this approach can free up research staff members from having to conduct the tACS sessions, thereby substantially increasing the intervention’s scalability and access. High adherence levels observed throughout the Acute Phase and Taper Phase provide evidence for the feasibility of the HB-tACS approach. The 125% adherence during the Taper Phase indicates that study companions were administering more sessions than the minimum amount requested by research staff. These findings are remarkable given that better treatment outcomes depend on higher levels of adherence and considering that individuals with chronic conditions frequently lose interest in following their prescribed treatment plan (Brown and Bussell, 2011).

All eight participants showed improvement in memory over the course of the trial. The improvement in memory at the end of the Acute Phase (7.38 points in the MIS score) was sustained 3 months later after the Hiatus Phase (7.00 points) and 6 months later after the Taper Phase (4.63 points). The episodic memory enhancement that was maintained at the end of the Taper Phase relative to baseline is clinically encouraging given the progressive nature of episodic memory deficits in AD. However, given the lack of control group in this study, it is impossible to determine whether these effects were due to the stimulation or due to some other element of trial participation. Importantly, potential practice effects on the MIS test were minimized using different memory stimuli during each iteration of the test. In fact, if memory from a previous run of the test would have influenced performance, it likely will have been in a negative fashion through proactive interference. However, it is possible that practice effects could have had a potential impact. It is worth noting that the MoCA scores remained the same even when MIS scores increased. This is in line with previous literature that suggests that targeting domains of cognitive functioning with non-invasive brain stimulation may be a net zero-sum game (Brem et al., 2014). This is something that merits further investigation in larger, well controlled trials.

Finally, the observed EEG changes in the theta/gamma ratio may have a potential relation to the observed behavioral improvement and might indicate neurophysiological changes induced by repeated tACS exposure. Specifically, it has been shown that increased theta/gamma ratio is associated with memory impairment in AD (Moretti et al., 2009) and it is predictive of progression from MCI to AD (Moretti et al., 2011). Our results could potentially indicate a relation between a decrease in theta/gamma ratio and improvement in memory function. However, these are very preliminary results, and our small sample size for the EEG portion of this study makes it difficult to draw any robust conclusions. Further investigation is required to understand the relation between the behavioral and neurophysiological effects of HB-tACS targeted at the AG.

As a feasibility trial testing a novel memory-focused intervention for AD, our study has relevant limitations. First, the sample size is small. A partial explanation for this is that the novelty of this intervention approach required a preliminary pilot study. However, this small sample does limit the generalizability of the feasibility findings for this intervention. Secondly, as the first study of its kind, it was impractical to enroll a control group of AD patients who did not receive tACS or that received sham tACS. Therefore, it is impossible to account for potential placebo effects. Thirdly, the methodology for measuring changes in memory functioning was limited to a singular index. Future studies should aim to encompass a larger breadth of memory measurements to build a more well-rounded idea of how memory was impacted. Fourthly, the results of the EEG source localization are constrained by the elimination of a large number of electrodes due to noise, and the EEG was only assessed in a subset of participants, a too small sample to allow any conclusions to be drawn. Despite these limitations, this study provides relevant data for future tACS investigations in AD. Future studies should build off the present pilot study and aim to enroll higher numbers of patients and include appropriate controls to test clinical efficacy.

## Conclusion

This pilot study shows the feasibility and preliminary efficacy of a novel, home-based, remotely supervised, study companion-led, multi-channel neuromodulation intervention for older adults with AD. We observed an improvement in memory scores throughout the duration of the intervention while global cognition was relatively stable, indicating that future interventions might benefit from multifocal neuromodulation targeting multiple cognitive domains. Future additional randomized controlled studies will be required to determine the efficacy of HB-tACS intervention. Our findings, which show safety and high adherence to this intervention are encouraging for continuing research with home-based neuromodulatory interventions for memory functioning in AD. Further, the preliminary evidence about memory improvements and potential decreases in the theta/gamma ratio merit larger and ultimately better controlled trials to more definitively determine the effect of tACS on memory functioning and on the underlying brain activity in AD. Finally, the differential effects on memory and global cognition should be further explored by, for example, administering complex neuromodulation interventions that target multiple cognitive domains simultaneously.

## Data availability statement

The raw data supporting the conclusions of this article will be made available by the authors, without undue reservation.

## Ethics statement

This study involving human participants was approved and monitored by an independent Institutional Review Board (Advarra, Inc.). The patients/participants provided their written informed consent to participate in this study.

## Author contributions

DC: conceptualization and design of the study, analysis and interpretation of data, writing of the original manuscript draft, reviewing and editing, and supervision. RF: acquisition of data, writing of the original draft, reviewing and editing, figures preparation, and editing. TB: acquisition of data, writing of the original manuscript draft, and reviewing and editing. WY and NL: acquisition of data, and writing—review and editing. GC: writing—review and editing. RP-A: analysis of data, interpretation of data, and writing—review and editing. DB-F: interpretation of data, and writing—review and editing. BM: conceptualization and design of the study, interpretation of data, and writing—review and editing. AP-L: conceptualization and design of the study, interpretation of data, writing of the original manuscript draft, reviewing and editing of manuscript, and supervision. All authors contributed to the article and approved the submitted version.

## References

[B1] Abraham M. Seidenberg M. Kelly D. A. Nielson K. A. Woodard J. L. Carson Smith J. (2020). Episodic memory and hippocampal volume predict 5-year mild cognitive impairment conversion in healthy apolipoprotein ε4 carriers. *J. Int. Neuropsychol. Soc.* 26 733–738. 10.1017/s1355617720000181 32131913 PMC7423643

[B2] Adaikkan C. Middleton S. J. Marco A. Pao P.-C. Mathys H. Kim D. N.-W. (2019). Gamma entrainment binds higher-order brain regions and offers neuroprotection. *Neuron* 102 929–943.e8. 10.1016/j.neuron.2019.04.011 31076275 PMC6697125

[B3] Alzheimer’s Association (2022). *2022 Alzheimer’s disease facts and figures.* Chicago, IL: Alzheimer’s Association. 10.1002/alz.12638 35289055

[B4] Antal A. Alekseichuk I. Bikson M. Brockmöller J. Brunoni A. R. Chen R. (2017). Low intensity transcranial electric stimulation: Safety, ethical, legal regulatory and application guidelines. *Clin. Neurophysiol.* 128 1774–1809. 10.1016/j.clinph.2017.06.001 28709880 PMC5985830

[B5] Antal A. Paulus W. (2013). Transcranial alternating current stimulation (tACS). *Front. Hum. Neurosci.* 7:317. 10.3389/fnhum.2013.00317 23825454 PMC3695369

[B6] Avgerinos K. I. Ferrucci L. Kapogiannis D. (2021). Effects of monoclonal antibodies against amyloid-β on clinical and biomarker outcomes and adverse event risks: A systematic review and meta-analysis of phase III RCTs in Alzheimer’s disease. *Ageing Res. Rev.* 68:101339. 10.1016/j.arr.2021.101339 33831607 PMC8161699

[B7] Babiloni C. Lizio R. Marzano N. Capotosto P. Soricelli A. Triggiani A. I. (2016). Brain neural synchronization and functional coupling in Alzheimer’s disease as revealed by resting state EEG rhythms. *Int. J. Psychophysiol.* 103 88–102. 10.1016/j.ijpsycho.2015.02.008 25660305

[B8] Benussi A. Cantoni V. Cotelli M. S. Cotelli M. Brattini C. Datta A. (2021). Exposure to gamma tACS in Alzheimer’s disease: A randomized, double-blind, sham-controlled, crossover, pilot study. *Brain Stimul.* 14 531–540. 10.1016/j.brs.2021.03.007 33762220

[B9] Benussi A. Cantoni V. Grassi M. Brechet L. Michel C. M. Datta A. (2022). Increasing brain gamma activity improves episodic memory and restores cholinergic dysfunction in Alzheimer’s disease. *Ann. Neurol.* 92 322–334. 10.1002/ana.26411 35607946 PMC9546168

[B10] Brem A.-K. Fried P. J. Horvath J. C. Robertson E. M. Pascual-Leone A. (2014). Is neuroenhancement by noninvasive brain stimulation a net zero-sum proposition? *NeuroImage* 85 1058–1068. 10.1016/j.neuroimage.2013.07.038 23880500 PMC4392930

[B11] Brown M. T. Bussell J. K. (2011). Medication adherence: WHO cares? *Mayo Clin. Proc.* 86 304–314. 10.4065/mcp.2010.0575 21389250 PMC3068890

[B12] Cappon D. den Boer T. Jordan C. Yu W. Lo A. LaGanke N. (2022). Safety and feasibility of tele-supervised home-based transcranial direct current stimulation for major depressive disorder. *Front. Aging Neurosci.* 13:765370. 10.3389/fnagi.2021.765370 35185515 PMC8849231

[B13] Cappon D. D’Ostilio K. Garraux G. Bisiacchi P. Rothwell J. (2015). Cortical modulation of automatic facilitation and inhibition by 10 Hz and 20 Hz transcranial alternating current stimulation (tACS). *Brain Stimulation* 8:356. 10.1016/j.brs.2015.01.149 27038707

[B14] Cappon D. D’Ostilio K. Garraux G. Rothwell J. Bisiacchi P. (2016). Effects of 10 Hz and 20 Hz transcranial alternating current stimulation on automatic motor control. *Brain Stimulation* 9, 518–524. 10.1016/j.brs.2016.01.001 27038707

[B15] Charvet L. Shaw M. Haider L. Melville P. Krupp L. (2015). Remotely-delivered cognitive remediation in multiple sclerosis (MS): Protocol and results from a pilot study. *Multiple Scler. J. Exp. Transl. Clin.* 1:205521731560962. 10.1177/2055217315609629 28607707 PMC5433334

[B16] de Cheveigné A. (2020). ZapLine: A simple and effective method to remove power line artifacts. *NeuroImage* 207:116356. 10.1016/j.neuroimage.2019.116356 31786167

[B17] Delorme A. Makeig S. (2004). EEGLAB: An open source toolbox for analysis of single-trial EEG dynamics including independent component analysis. *J. Neurosci. Methods* 134 9–21. 10.1016/j.jneumeth.2003.10.009 15102499

[B18] den Boer T. Fox R. Yu W. LaGanke N. Manor B. Pascual-Leone A. (2022). “A remote training and supervision program from transcranial electrical stimulation in the home setting,” in *Poster at the Cognitive Neuroscience Society Annual Meeting*, (San Francisco, CA). 10.1177/1357633X19861830

[B19] Desikan R. S. Ségonne F. Fischl B. Quinn B. T. Dickerson B. C. Blacker D. (2006). An automated labeling system for subdividing the human cerebral cortex on MRI scans into gyral based regions of interest. *NeuroImage* 31 968–980. 10.1016/j.neuroimage.2006.01.021 16530430

[B20] El Haj M. Roche J. Gallouj K. Gandolphe M.-C. (2017). Autobiographical memory compromise in Alzheimer’s disease: A cognitive and clinical overview. *Gériatrie Psychol. Neuropsychiatr. Viellissement* 15 443–451. 10.1684/pnv.2017.0704 29187335

[B21] Etter G. van der Veldt S. Manseau F. Zarrinkoub I. Trillaud-Doppia E. Williams S. (2019). Optogenetic gamma stimulation rescues memory impairments in an Alzheimer’s disease mouse model. *Nat. Commun.* 10:5322. 10.1038/s41467-019-13260-9 31757962 PMC6876640

[B22] Hampel H. Mesulam M.-M. Cuello A. C. Farlow M. R. Giacobini E. Grossberg G. T. (2018). The cholinergic system in the pathophysiology and treatment of Alzheimer’s disease. *Brain* 141 1917–1933. 10.1093/brain/awy132 29850777 PMC6022632

[B23] Herrmann C. S. Rach S. Neuling T. Strüber D. (2013). Transcranial alternating current stimulation: A review of the underlying mechanisms and modulation of cognitive processes. *Front. Hum. Neurosci.* 7:279. 10.3389/fnhum.2013.00279 23785325 PMC3682121

[B24] Humphreys G. F. Lambon Ralph M. A. Simons J. S. (2021). A unifying account of angular gyrus contributions to episodic and semantic cognition. *Trends Neurosci.* 44 452–463. 10.1016/j.tins.2021.01.006 33612312

[B25] Iaccarino H. F. Singer A. C. Martorell A. J. Rudenko A. Gao F. Gillingham T. Z. (2016). Gamma frequency entrainment attenuates amyloid load and modifies microglia. *Nature* 540 230–235. 10.1038/nature20587 27929004 PMC5656389

[B26] Julayanont P. Brousseau M. Chertkow H. Phillips N. Nasreddine Z. S. (2014). Montreal Cognitive Assessment Memory Index Score (MoCA-MIS) as a predictor of conversion from mild cognitive impairment to Alzheimer’s disease. *J. Am. Geriatr. Soc.* 62 679–684. 10.1111/jgs.12742 24635004

[B27] Kim J. Kim H. Jeong H. Roh D. Kim D. H. (2021). tACS as a promising therapeutic option for improving cognitive function in mild cognitive impairment: A direct comparison between tACS and tDCS. *J. Psychiatr. Res.* 141 248–256. 10.1016/j.jpsychires.2021.07.012 34256276

[B28] Koenig T. Prichep L. Dierks T. Hubl D. Wahlund L. O. John E. R. (2005). Decreased EEG synchronization in Alzheimer’s disease and mild cognitive impairment. *Neurobiol. Aging* 26 165–171. 10.1016/j.neurobiolaging.2004.03.008 15582746

[B29] Kybic J. Clerc M. Abboud T. Faugeras O. Keriven R. Papadopoulo T. (2005). A common formalism for the Integral formulations of the forward EEG problem. *IEEE Trans. Med. Imaging* 24 12–28. 10.1109/tmi.2004.837363 15638183

[B30] Lei L. Lam B. Lai D. Bai X. Li J. Zou Z. (2022). Stability of montreal cognitive assessment in individuals with mild cognitive impairment: Potential influence of practice effect. *J. Alzheimers Dis.* 87 1401–1412. 10.3233/JAD-220003 35431252

[B31] Luu P. Ferree T. (2005). Determination of the geodesic sensor nets’ average electrode positions and their 10 – 10 international equivalents. Technical Note. Available online at: https://www.researchgate.net/publication/266609828_Determination_of_the_Geodesic_Sensor_Nets%27_Average_Electrode_Positions_and_Their_10_-_10_International_Equivalents (accessed September 15, 2020).

[B32] Martorell A. J. Paulson A. L. Suk H.-J. Abdurrob F. Drummond G. T. Guan W. (2019). Multi-sensory gamma stimulation ameliorates Alzheimer’s-associated pathology and improves cognition. *Cell* 177 256–271.e22. 10.1016/j.cell.2019.02.014 30879788 PMC6774262

[B33] Mazziotta J. C. Toga A. W. Evans A. Fox P. Lancaster J. (1995). A probabilistic atlas of the human brain: Theory and rationale for its development. *NeuroImage* 2 89–101. 10.1006/nimg.1995.1012 9343592

[B34] Moretti D. V. Fracassi C. Pievani M. Geroldi C. Binetti G. Zanetti O. (2009). Increase of theta/gamma ratio is associated with memory impairment. *Clin. Neurophysiol.* 120 295–303. 10.1016/j.clinph.2008.11.012 19121602

[B35] Moretti D. V. Frisoni G. B. Fracassi C. Pievani M. Geroldi C. Binetti G. (2011). MCI patients’ EEGs show group differences between those who progress and those who do not progress to AD. *Neurobiol. Aging* 32 563–571. 10.1016/j.neurobiolaging.2009.04.003 20022139

[B36] Nasreddine Z. S. Phillips N. A. Bedirian V. Charbonneau S. Whitehead V. Collin I. (2005). The montreal cognitive assessment, MoCA: A brief screening tool for mild cognitive impairment. *J. Am. Geriatr. Soc.* 53 695–699. 10.1111/j.1532-5415.2005.53221.x 15817019

[B37] Oh H. Madison C. Villeneuve S. Markley C. Jagust W. J. (2013). Association of gray matter atrophy with age, β-amyloid, and cognition in aging. *Cereb. Cortex* 24 1609–1618. 10.1093/cercor/bht017 23389995 PMC4014182

[B38] Palop J. J. (2020). Network abnormalities and interneuron dysfunction in Alzheimer’s disease. *Alzheimers Dement.* 16:e040396. 10.1002/alz.040396 PMC8162106 27829687

[B39] Palop J. J. Mucke L. (2016). Network abnormalities and interneuron dysfunction in Alzheimer disease. *Nat. Rev. Neurosci.* 17 777–792. 10.1038/nrn.2016.141 27829687 PMC8162106

[B40] Ruffini G. Fox M. D. Ripolles O. Miranda P. C. Pascual-Leone A. (2014). Optimization of multifocal transcranial current stimulation for weighted cortical pattern targeting from realistic modeling of electric fields. *NeuroImage* 89 216–225. 10.1016/j.neuroimage.2013.12.002 24345389 PMC3944133

[B41] Salmelin R. Baillet S. (2009). Electromagnetic brain imaging. *Hum. Brain Mapp.* 30 1753–1757. 10.1002/hbm.20795 19378271 PMC6870961

[B42] Sperling R. A. Laviolette P. S. O’Keefe K. O’Brien J. Rentz D. M. Pihlajamaki M. (2009). Amyloid deposition is associated with impaired default network function in older persons without dementia. *Neuron* 63 178–188. 10.1016/j.neuron.2009.07.003 19640477 PMC2738994

[B43] Tadel F. Bock E. Niso G. Mosher J. C. Cousineau M. Pantazis D. (2019). MEG/EEG group analysis with brainstorm. *Front. Neurosci.* 13:76. 10.3389/fnins.2019.00076 30804744 PMC6378958

[B44] van de Mortel L. A. Thomas R. M. van Wingen G. A. (2021). Grey matter loss at different stages of cognitive decline: A role for the thalamus in developing Alzheimer’s disease. *J. Alzheimers Dis.* 83 705–720. 10.3233/jad-210173 34366336 PMC8543264

[B45] van Dyck C. H. Swanson C. J. Aisen P. Bateman R. J. Chen C. Gee M. (2022). Lecanemab in early Alzheimer’s disease. *N. Eng. J. Med.* 388 9–21. 10.1056/nejmoa2212948 36449413

[B46] Welch P. (1967). The use of fast Fourier transform for the estimation of power spectra: a method based on time averaging over short, modified periodograms. *IEEE Trans. Audio Electroacoust.* 15, 70–73. 10.1109/TAU.1967.1161901

[B47] Wong A. Nyenhuis D. Black S. E. Law L. S. N. Lo E. S. K. Kwan P. W. L. (2015). Montreal cognitive assessment 5-minute protocol is a brief, valid, reliable, and feasible cognitive screen for telephone administration. *Stroke* 46 1059–1064. 10.1161/strokeaha.114.007253 25700290 PMC4373962

[B48] World Health Organization (2022). *Dementia.* Geneva: World Health Organization.

[B49] Zaehle T. Rach S. Herrmann C. S. (2010). Transcranial alternating current stimulation enhances individual alpha activity in human EEG. *PLoS One* 5:e13766. 10.1371/journal.pone.0013766 21072168 PMC2967471

[B50] Zhou D. Li A. Li X. Zhuang W. Liang Y. Zheng C.-Y. (2022). Effects of 40 Hz transcranial alternating current stimulation (tACS) on cognitive functions of patients with Alzheimer’s disease: A randomised, double-blind, sham-controlled clinical trial. *J. Neurol. Neurosurg. Psychiatry* 93 568–570. 34764150 10.1136/jnnp-2021-326885

